# HIV sexual risk behaviors in youth 15-24 years of age in Cali, Colombia: Do differences exist among neighborhoods?

**Published:** 2013-06-30

**Authors:** Sandra L Girón, Hannia Palacio, Julio C Mateus

**Affiliations:** a Universidad del Valle, FES Foundation, School of Public Health, Cali., Division of Health, FES Foundation, Cali; School of Public Health, Universidad del Valle, Cali. E-mail:, jcmateus@fundacionfes.org; b Division of Health, FES Foundation, Cali. E-mail: sandragiron@fundacionfes.org

**Keywords:** Acquired Immunodeficiency Syndrome, HIV, sexual behaviors, and sexual partners

## Abstract

**Introduction:**

HIV/AIDS is a global health priority. About 40% of new infections occur among heterosexual youth by means of sexual contact. In Cali, district 13, 15 and 20 account for 11.5% of the prevalent cases and 18.0% of incident cases.

**Objective:**

To establish differences in risk behaviors for HIV among young people 15-24 yrs of age from two areas of Cali, Colombia.

**Methods:**

We carried out a cross-sectional study among young people between 15 and 24 yrs of age in these districts. The selection was done with a two-stage probability sampling. We estimated the prevalence of sexual relationships without condom usage, sex with multiple partners, and sex under the effects of alcohol and through logistical regression we identified the related factors.

**Results:**

In district 13, 15 and 20, the prevalence of unprotected sexual relationships in the last 12 months and the prevalence of sex with two or more partners was 70%; and 38% of young people had sex under the effects of alcohol. In both areas, the intention was positively related to the risk behaviors. We found socio-demographic factors, intentions, and beliefs that increase the opportunity to display these behaviors. The effect of these factors differs by district.

**Conclusions:**

We observed a high prevalence of risk behaviors for HIV related to socio-demographic factors, intentions and beliefs that warrant interventions appropriate for local realities.

## Abstract (es)

**Introducción:**

El VIH/SIDA es una prioridad en salud mundial. Alrededor de 40% de las infecciones nuevas se producen por contacto sexual en jóvenes heterosexuales. En Cali, las comunas 13, 15 y 20 agrupan 11,5% de casos prevalentes y 18.0% de los incidentes.

**Objetivo:**

Establecer diferencias en comportamientos sexuales de riesgo para VIH entre jóvenes de 15 a 24 años de dos áreas de la Ciudad de Cali. Métodos: Se realizó un estudio transversal en jóvenes de 15 a 24 años de las comunas 13 y 15 y de la comuna 20. Se realizó muestreo probabilístico bietápico. Se midieron las prevalencias de relaciones sexuales sin condón, con múltiples compañeros y bajo efectos del licor. Con regresión logística se identificaron los factores relacionados a estos comportamientos.

**Resultados:**

La prevalencia de relaciones sin condón en los últimos 12 meses y de relaciones con múltiples compañeros en las comunas 13 y 15 y en la 20 fue 70%. La prevalencia de relaciones sexuales bajo efectos del licor fue 38%. En ambos grupos de comunas, la intención de ejecutar el comportamiento de riesgo se relacionó positivamente con los comportamientos de interés. Además, se encontraron factores sociodemográficos, creencias normativas, creencias de control, motivación para cumplir y de poder percibido relacionados con los comportamientos de riesgo, los cuales tienen efecto diferente para cada grupo de comunas.

**Conclusiones:**

Se observaron altas prevalencias de comportamientos de riesgo frente a VIH, relacionadas con factores sociodemográficos, intenciones, creencias y percepciones, que requieren ser intervenidos de acuerdo a las realidades del entorno.

## Introduction

HIV/AIDS is one of the main health challenges worldwide^1^. About 40% of new infections occur in young people through heterosexual contact. For this reason, UNAIDS recognizes youth between 15 and 24 yrs as population highly vulnerable to infection, given the opportunity to participate in high risk behaviors, such as unprotected sex, sex with multiple partners and sex under effects of alcohol^1^ ^,^ ^2^.

Several studies report that the HIV epidemic is the result of biological factors, but also behavioral factors that increase the likelihood of acquiring the infection. Examples include unprotected sexual relations, long-term concurrent multiple sexual partnerships, and the initiation of sex at an early age^3^ ^,^ ^4^. It also recognizes that there are underlying cultural and socioeconomic factors for each population, such as power differences in intimate relationships, sexual rights, cultural expectations of males and females and economic inequality that contribute to the growth of the epidemic^5^ ^,^ ^6^.

Among Colombian youth there are sexual risk behaviors that increase the vulnerability to HIV^7^. The onset of sexual relations is reported to be at an increasingly early age (14 years average, range 5-21), condom usage is reported only among 25% of youth, and there are reports of more than one sexual partner in over 40% of cases^8^. In Cali, young people between the ages of 15 to 24 accounts for 30% of the population, 23% of new HIV infections, and 98% of cases with reported sexual transmission^8^ ^,^ ^9^. Between 50 to 54% of youth report not using condoms on their first sexual experience or on their last one, and 63% report an inconsistent use of condoms^10^. In addition, 72% of youth report having sex with multiple partners and 48% report having had sex under the influence of alcohol^10^ ^,^ ^11^.

In Cali, the distribution of incidence and prevalence cases has been identified as differing among the political-administrative areas that divide the city, labeled communes. In particular, communes 13, 15 and 20 accounted for 11.5% of prevalence cases and 18.0% of incidence cases^12^. This situation warrants arriving at estimates of the prevalence of risk behaviors in this population and determining whether the risk and prevalence factors differ between areas of the city, taking into account that the field of juvenile sexuality also requires the design and development of interventions according to local realities^13^ ^,^ ^14^.

Although interventions in health promotion and prevention of HIV / AIDS in young people were addressed through the health and education sectors, in most cases the strategies implemented have been built without recognizing local differences, have not relied on a theoretical basis of behavioral change, have not fully taken into account research results and they have not been evaluated^15^. Therefore, there is a clear need for studies to identify the factors that help explain the expression of behaviors that increase the risk of acquiring HIV.

This study is the initial phase of a larger project that aims to develop and evaluate a community intervention directed toward modulating sexual risk behaviors for HIV infection among 15 to 24 year olds from the lower socioeconomic strata of Cali. This project involves the communities identified as communes 13, 15 and 20 which have had a high reported level of HIV cases in recent years. Additionally, these communes differ greatly in their ethnic and geographical contexts in that they were formed mainly of migrants from the Pacific region (communes 13 and 15) and from the Andean region (commune 20) of South-Western Colombia^16^. In particular, commune 20 was established during the 1950s while communes 13 and 15 were established in the early 1980s. Commune 20 is mainly composed of descendants of Mestizo origin while the latter two are principally composed by those of African descent. Under these conditions, the project sought to establish whether the interventions within the context of the city should differ according to local micro-contexts. For these reasons, this prevalence study has a dual purpose: to serve as a baseline for evaluating the effectiveness of interventions aimed at changing risky sexual behavior in young people and to establish whether at risk sexual behaviors have different expressions within the urban local contexts.

## Material and Methods

A cross-sectional study was carried out concerning the prevalence of HIV risk behaviors and related factors in communes 20, 13 and 15 of the city of Cali, Colombia. The selection of young people was carried out through independent, probabilistic, two-stage samplings for communes 13, 15 and 20. Blocks were selected and a subsequent household census was made to select a representative sampling that depended on the number of homes on the block. Subsequently we selected young individuals of both sexes from 15 to 24 years of age from these families who were residents in each of the selected households.

Collection of information started with an instrument developed for the Municipal Surveillance System for Risk Behaviors with HIV/AIDS for Youth from Cali in which is included in the guidelines of UNAIDS, the Theory of Planned Behavior^17^, and the results of cognitive tests to ensure understanding of language and to resolve situations that might affect the validity of the measurement^18^. The information was collected by three field teams previously trained to standardize operating procedures of the study.

To obtain data from the participants, letters were sent to the heads of household that explained the nature and scope of the research. Once young people were selected from these household to be surveyed, the procedures were explained to them, and they were asked for their written consent for participation. The data were entered into a database designed with Epi-info 2008 (version 3.5.1). Quality control was performed by comparing 10% of the registrants with the written forms while allowing for an acceptable minimum error rate of 2% for each question. Discordant data were identified and corrected. Subsequently, the database was exported to Stata(r) (version 10.0) where additional tabulations were performed to identify implausible, missing or inconsistent data.

Due to differences in the final probability for selection of the youth, the results were weighted by the basic factor of expansion, constituted by the inverse probability of selection of the block, the home and the probability of response^19^. The prevalence of sex without a condom was estimated, as well as sex with multiple partners and sex under the influence of alcohol, with respective confidence intervals at 95%. For variables that assessed these constructs in more than two categories (e.g. how likely is it that a condom was used in all sexual relations? Very Likely, Likely and Unlikely), prevalence was estimated for each category and the categories were also grouped to confer risk of acquiring the infection (e.g., Little/Not possible). An analysis of each of the variables was performed using 2xn tables, and chi square tests were performed to assess the statistical significance.

The variables that had statistical significance less than 0.25 from the univariate analysis were included in multiple model construction. Subsequently, multiple logistical regressions were performed using 0.2 backward probabilities. After evaluating the presence of co-linearity (Spearman coefficients), the beliefs, perceptions and intentions regarding the mentioned potentially risky behaviors were measured. OR raw data were compared with the OR adjusted data to establish the presence of any signs of confusion or effect modification. In the final model obtained outliers were examined by calculating residuals and by graphical representation. On identifying variations greater than 10%, the plausibility of the data was evaluated in order to determine their retention or exclusion from the model. To evaluate the fit of the model the Hosmer-Lemeshow test was used and the lack of significance indicated a good fit to the model.The ethical aspects were approved by the Institutional Committee of FES and the Ethics Committee of the University of Valle

## Results

The collection of data was carried out between January 2008 and August 2009. The non-response rate was 21.24% for communes 13 and 15 and 16.09% for commune 20. 805 youth were selected from communes 13 and 15, while 696 were drawn from commune 20. Table 1 shows that for all communes surveyed more than half of the young people were male, 50% were 18 years of age or less, and most were students with incomplete secondary schooling. 

**Table 1:**
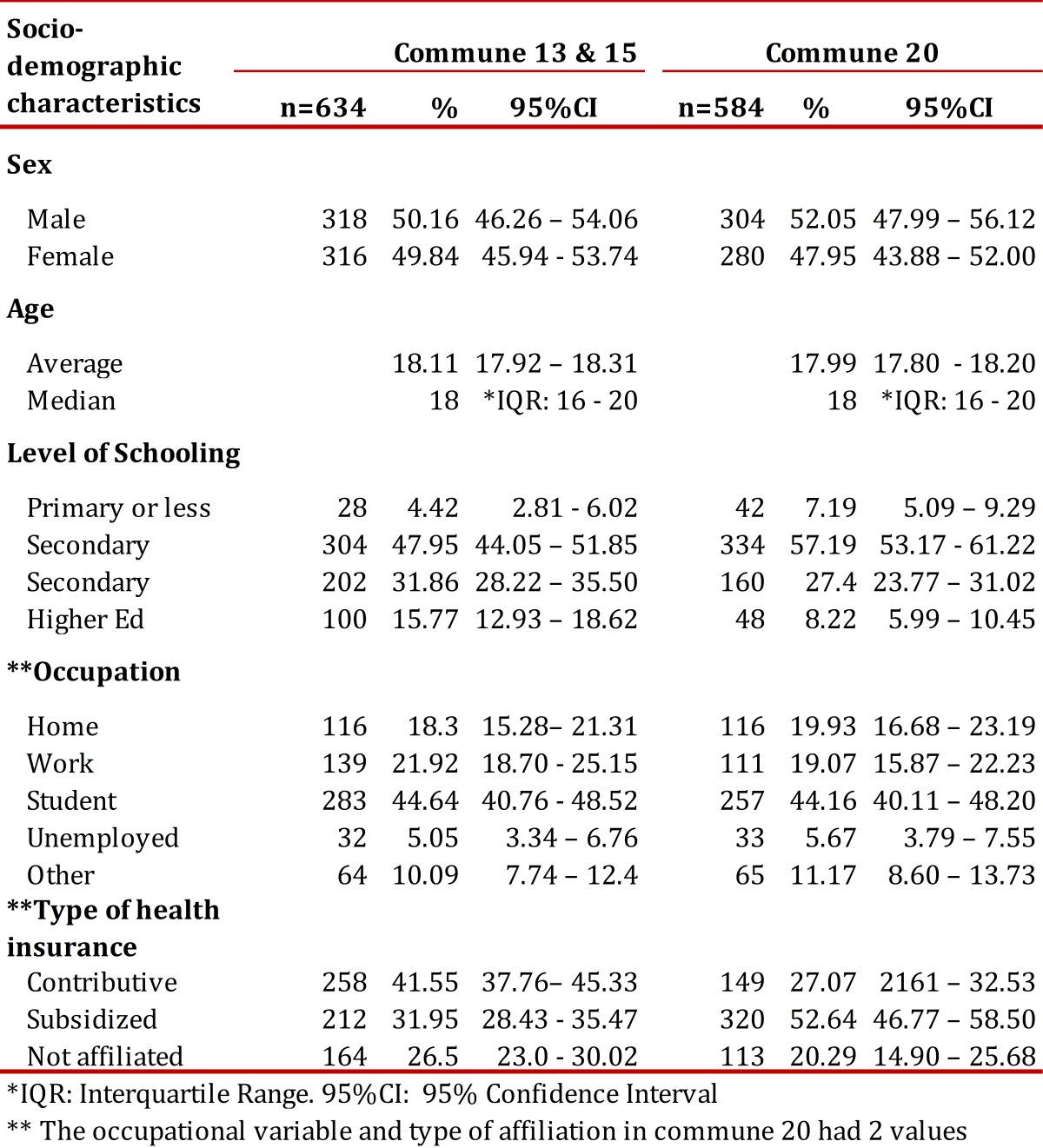
Socio-demographic characteristics of the youth surveyed, *IQR: Interquartile Range, ** The occupational variable and type of affiliation in commune 20 had 2 values omitted from the survey form.

It was estimated that for this population a high prevalence of risk behaviors for acquiring HIV would be found, along with similar magnitudes among the communes. The prevalence of sex without a condom at first intercourse, during the past 12 months, and sex with multiple partners was around 70%. In turn, it was found that about 60% of young people had reported sex under the influence of alcohol, and in almost 70% of cases use condoms was not reported (Table 2).

**Table 2.**
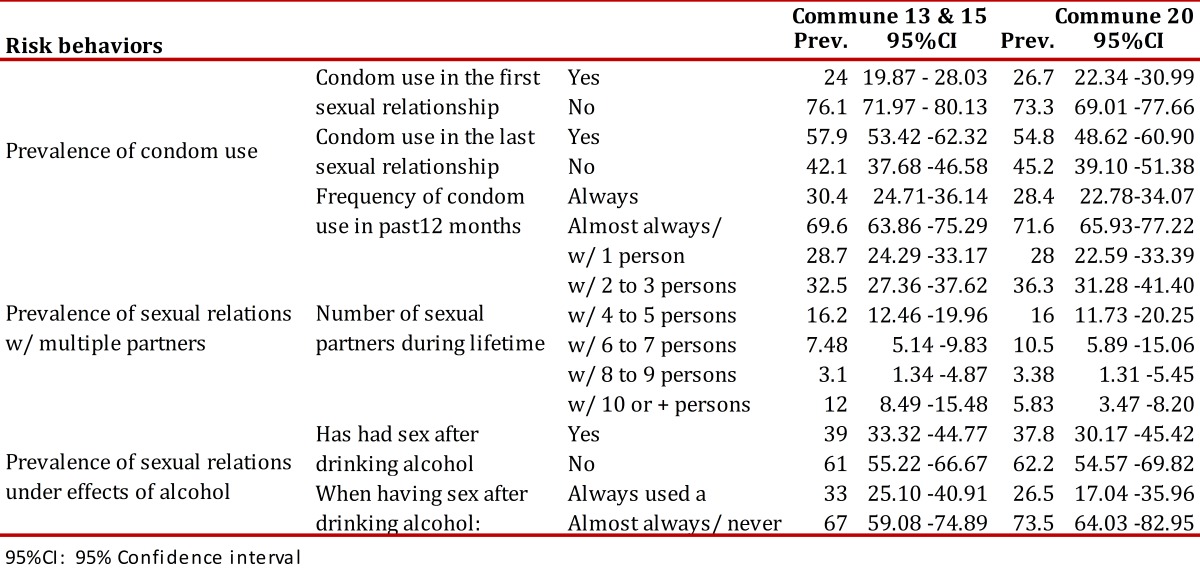
Prevalence of risk behaviors for HIV among youth from 15 to 24 years of age in Cali by commune.

With regard to factors related to the prevalence of sex without a condom in the past 12 months and during the last reported sexual relationship, it was found that in both groups of communes that low intention to use condoms increased chances of having had unprotected sex by at least three times (Tables 3 and 4).

**Table 3.**
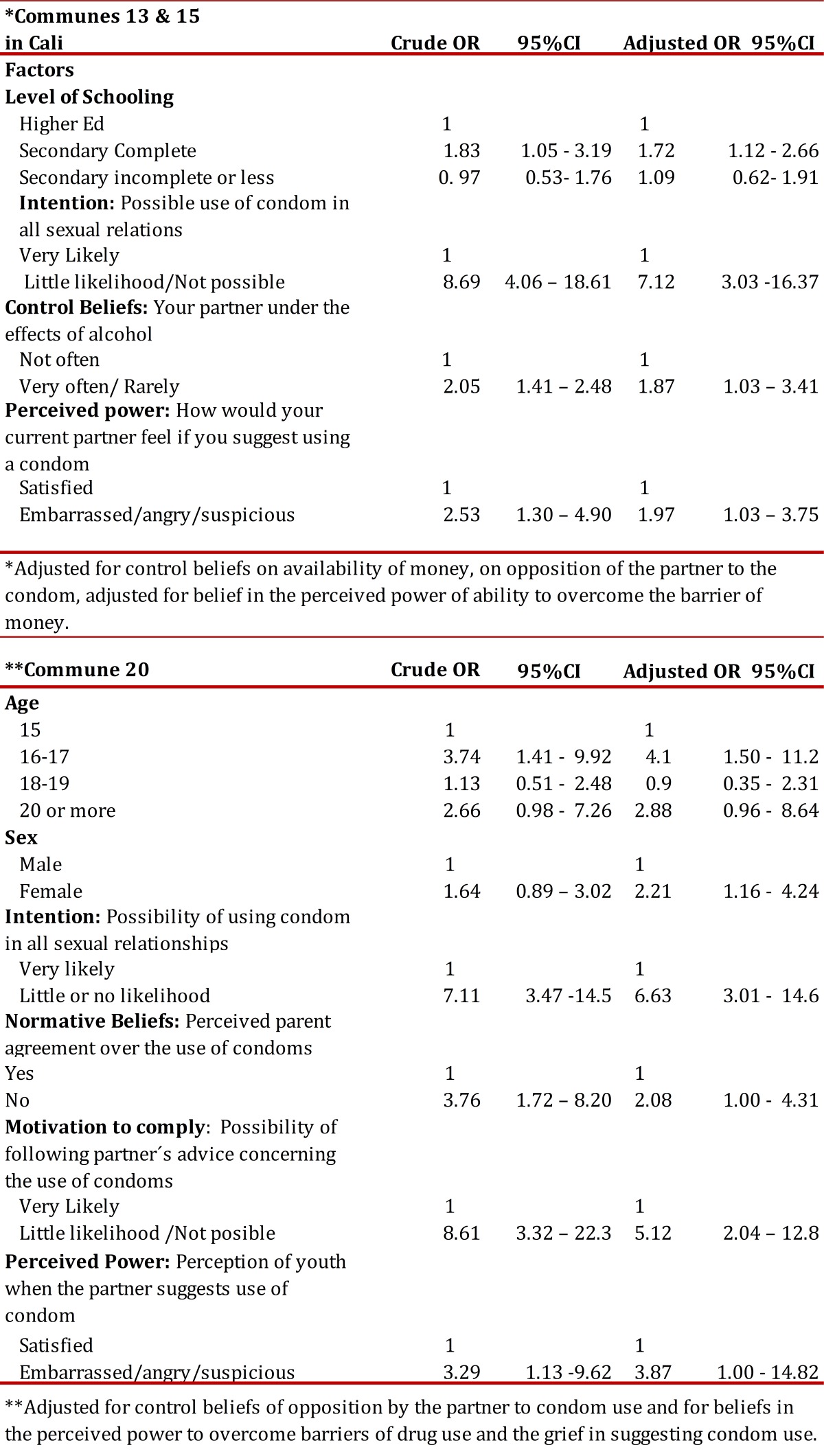
Multiple logistical analysis of factors related to use of condom in sexual relations during the past 12 months, by commune.**Adjusted for control beliefs of opposition by the partner to condom use and for beliefs in the perceived power to overcome barriers of drug use and the grief in suggesting condom use.

**Table 4.**
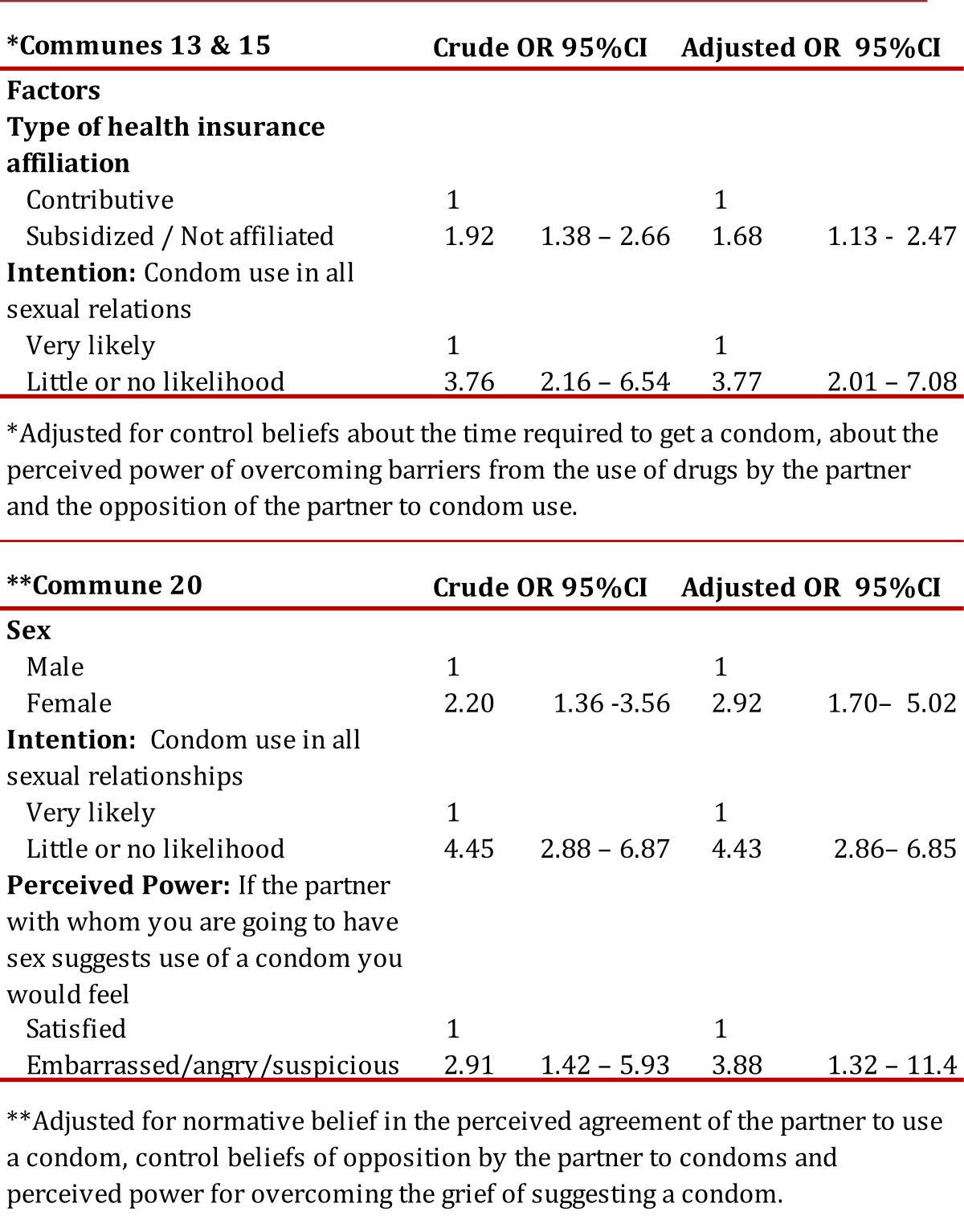
Multiple logistical analysis of factors related to condom use in the last sexual relationship, by commune. **Adjusted for normative belief in the perceived agreement of the partner to use a condom, control beliefs of opposition by the partner to condoms and perceived power for overcoming the grief of suggesting a condom.

With regard to factors related to the prevalence of sex without a condom in the past 12 months, it was found that in communes 13 and 15 the opportunity for this to happen was higher among those with incomplete secondary education, among those who often considered relations under the influence of liquor, and among those who perceived that the partner would not feel satisfied by suggested use of a condom (Table 3).

In turn, in commune 20 it was found that there was a greater chance of having sex without a condom in the past 12 months among youth of 16 to 17 years, among females, among those with perceived disapproval from the parent when faced with condom use, among those with a low probability of following the advice of the partner concerning condoms, and among those who believed that they would not be satisfied if the partner suggested condom use (Table 3).

With regard to factors related to the prevalence of not using a condom during the last reported sexual incident, it was found that the likelihood of this happening in communes 13 and 15 was 68% greater among those youth who reported having their health insurance subsidized by the government and among those without insurance affiliation. Moreover, in commune 20 it was found condom disuse was greater among women and among those who believed that the partner would not feel satisfied if condom use was suggested (Table 4). In reference to the factors related to the prevalence of sex with multiple partners, it was found that in all communes the chance of having more than one sexual partner increased between 2 and 3 times with the increasing age of the youth. In turn, in communes 13 and 15 women had 78% less of a chance of having had multiple sexual partners, a 97% greater chance among those who considered following the advice of a parent when facing this behavior, and a 79% greater chance of this happening among those who see it is as likely that they will have a new partner with someone that looks healthy (Table 5).

**Table 5:**
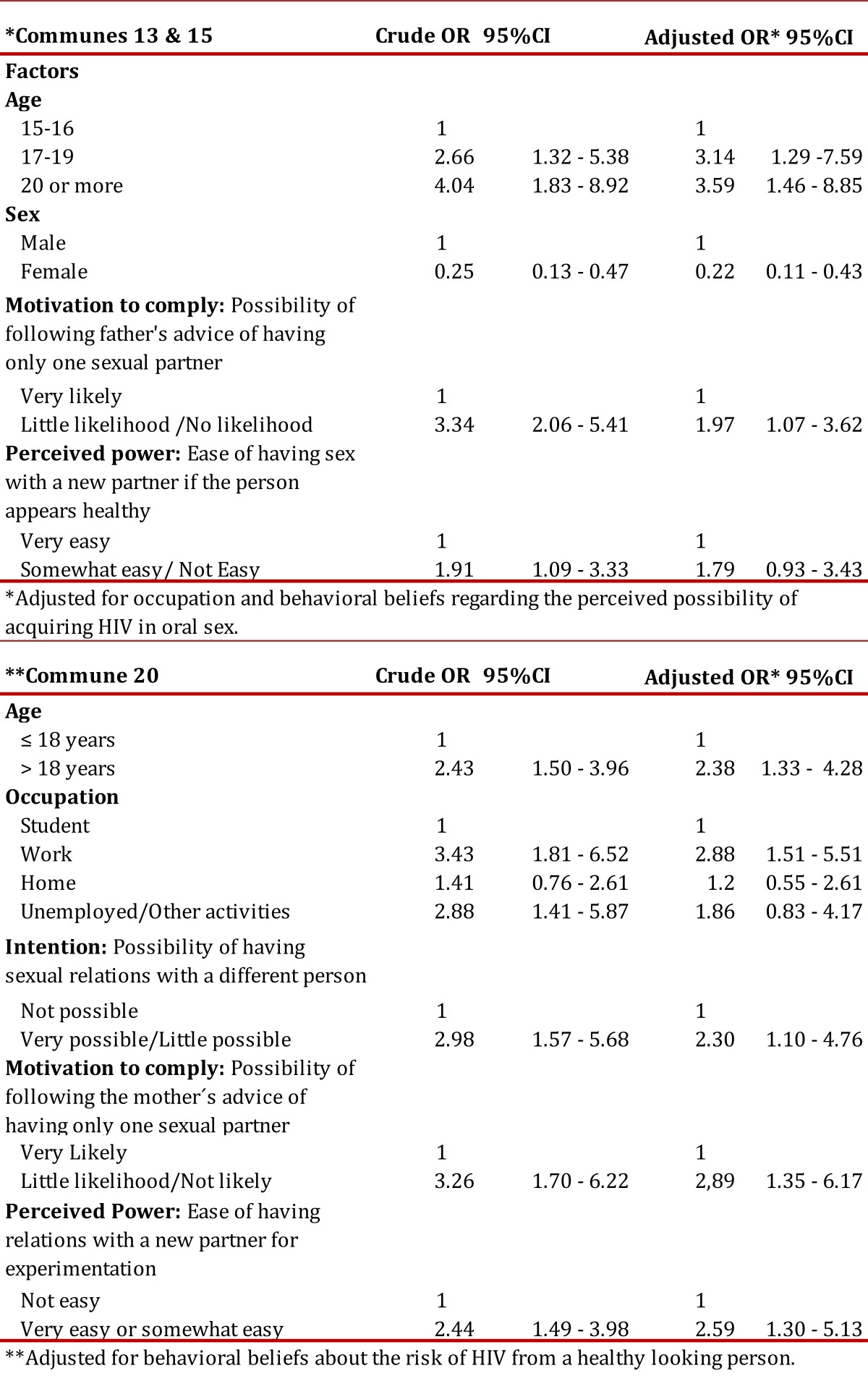
Logistical analysis of factors relating to the prevalence of sexual relations with multiple partners during a lifetime, by commune.**Adjusted for behavioral beliefs about the risk of HIV from a healthy looking person.

On the other hand, in commune 20 it was found that young people engaged in work, those with low intention of having only one sexual partner, those who believed it possible to follow the advice of the mother when dealing with this behavior, and those who believed that the desire to experiment made it easier to have a new partner were more than twice as likely to have multiple partners (Table 5).

Finally, concerning the factors related to the prevalence of relationships under the influence of liquor, it was found in all communes that with increased age there was more than twice the chance of having sex under the influence of liquor. Additionally, it was found that in these communities the opportunity for this behavior increased 6-fold among those with a positive intention of doing it. Specifically, for communes 13 and 15, it was found that the chance of having had relations under the influence of liquor increased among those who perceived that the partner would agree to have sex under the influence of liquor and among those who perceived it possible to follow the advice of friends when dealing with this behavior (Table 6).

**Table 6.**
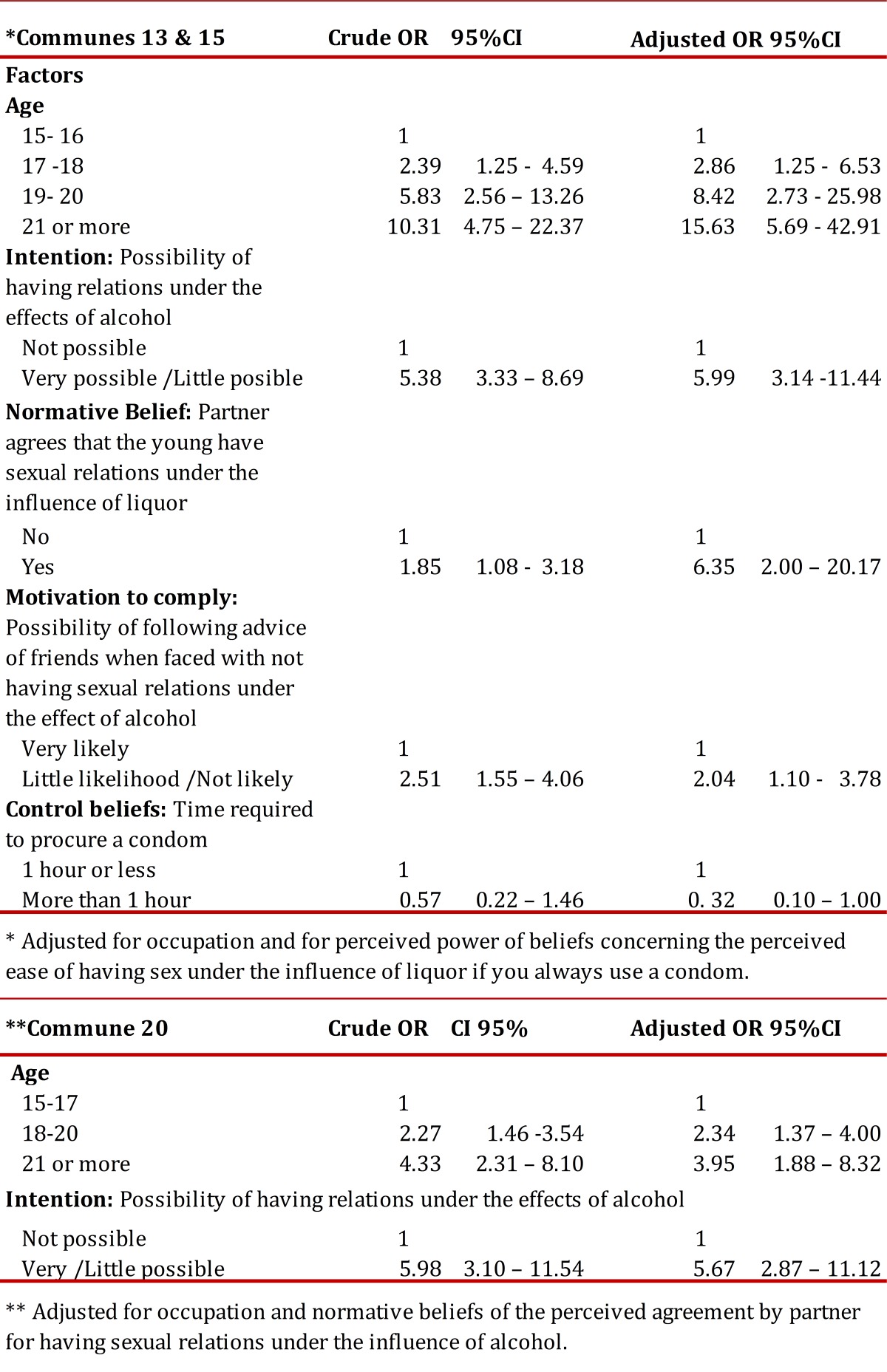
of sexual relations under the effects of alcohol, by commune. ** Adjusted for occupation and normative beliefs of the perceived agreement by partner for having sexual relations under the influence of alcohol. Multiple logistic analysis of factors related to the prevalence

There was no evidence of confounding phenomena or effect modifications in the multiple models present. There were no outliers found that influenced estimates and a good fit with the data was observed.

## Discussion

This research found a high prevalence of risk behaviors for acquiring HIV (sex without a condom, relations with multiple partners, and relationships under the influence of alcohol) and identified intervening factors that help minimize the risk of infection among youth in Cali.

In regard to unprotected sex, in both groups of communes it was observed that about 70% of the young participants did not use a condom with their first sexual intercourse, and did not always use it in the past 12 months. Further, over 40% reported not having used it during their last sexual intercourse. This high prevalence of sex without a condom is consistent with findings from other reported parts of Cali and Valle del Cauca where prevalence between 45% and 70% has been observed^7^ ^,^ ^9^. Also, the prevalence of condom use at last intercourse in the national context has been reported to be around 38%^7^ ^,^ ^20^, and among countries in Latin America and the Caribbean the prevalence is upwards of 80% ^21^ ^,^ ^22^.

In this study the high prevalence of sexual relations without condoms was found related to socio-demographic factors, intentions and beliefs that were similarly shared among the communes, but with differences noted. Specifically, it was observed that the low intention of using a condom resulted in a 3 to 7 time greater likelihood that the youth would not use a condom. According to the theory of planned behavior, intention is the best predictor of behavior. This finding is important in designing health interventions^23^. In addition, it has been reported that intention of using a condom as a predictor of condom use among young people has correlations above 45% ^23^.

Among the socio-demographic factors associated with condom use, a positive relationship was observed between this behavior and risk factors, such as being between 16 and 17 years old, female, having an incomplete secondary educational level and having state subsidized health insurance or no affiliation with the health system. These factors lead to increases of more than double the chance for not using condoms and they relate differently by each group of communes. This difference in the related factors creates an inequity in exposure to the virus, which is confirmed in several national and international studies reporting beliefs and cultural patterns of domination as risk factors in the most vulnerable groups that lead to inequalities in the negotiation of sexual and social relations^7^ ^,^ ^24^. Additionally, greater frequency of condom use in high and middle socioeconomic strata has been reported among those with higher levels of education and employment that allow for greater bargaining power in the relationship^7^. It is therefore important to develop strategies to strengthen the knowledge and bargaining power among vulnerable groups to overcome barriers and develop safe sexual behavioral patterns.

With respect to beliefs motivations and perceptions, an increase between 2 and 6 times was found for the chance of not using condoms in youth who often considered the partner under the influence of alcohol, among those who perceived parental disagreement when dealing with condom use, among those with low possibility of following advice from the partner when dealing with this behavior, and among those who perceived that he or the partner would feel dissatisfied by suggesting condom use. This greater chance of not using a condom shows deficiencies in the skills and knowledge needed for protection in this population group and the low possibility of protection and negotiation in the presence of liquor. Consistently, national and international studies have reported a relation between alcohol and the less frequent use of condoms^7^ ^,^ ^20^ ^.^

In addition, the importance of training parents, friends and the couple to start conversations when dealing with protective behaviors and overcoming cultural barriers, such as beliefs that prevent condom use among these most vulnerable groups has been reported^7^, ^20^ . On the other hand, the prevalence of sex with multiple partners was observed in both groups of communes of more than 70%, which when combined with the high prevalence of unprotected relations evidence an increased exposure to HIV risk. In this study this behavior was found associated with an increase in age, which tripled the chance of having multiple sexual partners. Similarly, several studies report associations three times greater between age and multiple sexual partners and have found that the age of first intercourse is a predictor of the number of partners^7^ ^,^ ^14^. According to UNAIDS, the prevalence of sex with multiple partners is 56%^1^. However, the same agency reported difficulties in consolidating its prevalence data as only 19 countries reported sufficient data for the estimation and it is recognized that the type of measurement varies according to age, sex and educational level of the young people^1^.While in communes 13 and 15 an increased chance of having sexual relations with multiple partners was observed among youth with little likelihood of following the advice of a parent when dealing with this behavior, in commune 20 this increase was seen among those with little possibility of following the advice of the mother. Specifically, those who did not consider following the advice of parents showed a 97% greater chance of having multiple partners.

This finding points out the influence of the concept of guides in the development of behavior. Consistently, several studies have reported a high prevalence of sex with multiple partners in youth with little communication with parents when dealing with sexual matters, which added to the perception of invulnerability when facing infection has generated greater exposure to the virus. Further, a relationship has been found between the quality and communicative attitude of parents and the development of safe sexual behavior^25^.

Additionally, in these communes a relationship was observed between the prevalence of sex with multiple partners, the sex of the participant, and with the possibility of following a parent´s advice of having only one sexual partner.

This relationship probably lies in reinforcing cultural factors for having more sexual partners in the male population, exposing them to increased risk of acquiring infection. This result is consistent with findings from the National Demographic and Health Survey that reported differences in bargaining power between men and women and the influence of cultural differences on sexual relationships^7^. Likewise, international estimations find more than twice the risk of having multiple sexual partners in the male population, which is consistent with HIV estimates for Latin America where only 35% of people living with HIV are women^1^.

Finally, a high prevalence of sex was observed under the influence of alcohol in communes 13, 15 and 20. About 38% of young people had sex after drinking liquor and only a third of them used condoms. This high prevalence was found to be related to age and to the intent of having sex under the influence of substances. Liquor consumption increases the possibility of producing risk behaviors that lead to greater exposure to HIV due to it decreasing the perception of risk and the possibility for negotiating safe sex^2^ ^,^ ^4^.Additionally, a relationship has been reported between alcohol consumption, intention to use this substance, and the development of risk behaviors such as sexual intercourse under the influence of alcohol.

These studies have found correlations on the order of 27% and show an increase in the chance of having unprotected sex and multiple sexual partners, as well as decreased bargaining power between partners due to aggression in youth under the influence of this substance and the justification of feeling stronger and extroverted when liquor is consumed^2^ ^,^ ^4^.

The findings of this research in addition to showing a high prevalence of risk behaviors in youth highlight that the factors related to condom use, to sex with multiple partners and to sex under the influence of liquor act differently among the communes, either by its magnitude or by the presence or absence of relationship. In this way it helps to operationalize national and international recommendations for designing interventions based on the local needs of young people, given that the above factors that influence the risk of acquiring HIV are different according to the segments of the population. Only then will an effective response to the HIV/AIDS be considered possible^2^.

## Conclusion

A high prevalence of HIV risk behaviors which need to be subjected to intervention were observed in young people between the ages of 15 and 24 years in communes 13, 15 and 20. Given that prevalence was found related to socio-demographic factors, intentions, beliefs and perceptions that differ among the communes both in magnitude and the direction of the relationship, the design of intervention strategies appropriate to the local realities of youth is recommended. The Theory of Planned Behavior allows an adequate approach for identifying intervening variables towards reducing the prevalence of these behaviors.
